# Between gait and cognition: an exploratory mixed-methods study of cognitive—motor dual-task profiles and lived experience

**DOI:** 10.3389/fpsyg.2026.1868678

**Published:** 2026-09-22

**Authors:** Fabien Buisseret, Frédéric Dierick, Eva Houdart, Florence Jonckheere, Samir Beggache, Jennifer Denis

**Affiliations:** 1 Laboratoire Forme et Fonctionnement Humain (FfH), CeREF – Santé, Haute Ecole Louvain en Hainaut, Montignies-sur-Sambre, Belgium; 2 Service de Physique Nucléaire et Subnucléaire, Research Institute for Complex Systems, Université de Mons, Mons, Belgium; 3 RehaLAB, Centre National de Rééducation Fonctionnelle et de Réadaptation–Rehazenter, Luxembourg City, Luxembourg; 4 Faculté des Sciences de la Motricité, UCLouvain, Ottignies-Louvain-la-Neuve, Belgium; 5 Unit of Clinical Psychology, University of Mons, Mons, Belgium

**Keywords:** adaptive behavior, cognitive load, explicitation interview, locomotion, multitasking

## Abstract

**Background:**

Cognitive-motor dual tasking is widely studied. However, the results remain heterogeneous depending on the type of tasks and individual psychological factors. To address this variability, dual-task cost (DTC) frameworks allow classification of behavioral profiles. This study aimed to propose a method designed to identify individual determinants of dual-task (DT) behavior by combining quantitative gait and cognitive measures with qualitative analyses of lived experience.

**Methods:**

Thirty-three young healthy adults performed Raven's Progressive Matrices Test (RPMT) and treadmill walking in both single-task and DT conditions. Participants were classified into DT profiles according to DTC. Statistical tests were performed to assess whether the DTCs differed from zero and whether they differed between participants who reported having answered some items randomly and those who did not. Explicitation interviews were then conducted and analyzed independently of participants' quantitative DT profiles. Qualitative findings were subsequently integrated with the quantitative profiles at the interpretive stage to characterize individual DT behaviors.

**Results:**

Dual tasking significantly reduced RPMT response time (*p* < 0.001) without affecting gait parameters. All four DT profiles were represented, and explicitation interviews revealed distinct experiential strategies within each profile.

**Discussion:**

Distress and cognitive overload characterized mutual interference, analytical or automatized strategies supported cognitive-priority trade-offs, and relaxed immersion and positive affect facilitated mutual facilitation. Gait-priority participants reported bodily immersion and sensory detachment.

**Conclusion:**

Our mixed-method approach may offer a transferable methodology for clinical contexts, where understanding patient-specific strategies is crucial for rehabilitation.

## 1 Introduction

Everyday mobility rarely occurs in isolation: People walk while conversing, navigating unfamiliar environments, or monitoring hazards. Performance under such dual-task (DT) conditions depends on how limited control resources are allocated between concurrent goals rather than on a fixed structural bottleneck (Wickens, 2008). While average group effects are often small or inconsistent, inter-individual variability is striking in DT since some people preserve or even improve performance, whereas others show deterioration on one or both tasks, see, e.g., the recent study by (Dierick et al. 2025). To what extent are a motor and a cognitive task impaired or facilitated when performed at the same time? This issue is particularly relevant in healthcare. For example, in physiotherapy, DT is used to improve the functional rehabilitation of patients with locomotor or neurological disorders, see the reviews by Fritz et al. (2015); Ali et al. (2022); Tan et al. (2024). Note that cognitive-motor DT is not the only possible DT: Motor-motor and cognitive-cognitive DT can occur too. It has been shown that, in elderly subjects, cognitive-motor DT had more impact on gait features than the other types of DT (Nedovic et al., 2024; Nedović et al., 2025). This led the authors of these last studies to conclude that a gait assessment involving a DT paradigm in healthy elderly subjects may allow for an early identification of cognitive impairment.

Contemporary attentional control studies propose that affective states bias the balance between goal-directed and stimulus-driven attention (Eysenck et al., 2007). Under distress or worry, control resources tilt toward threat monitoring and stability maintenance; under challenge/eustress, resources are deployed more flexibly. In mobility, these biases map onto the posture-first principle (prioritizing stability when perceived risk is high) vs. goal-first policies that preserve cognitive performance even at potential motor cost (Woollacott and Shumway-Cook, 1990; Yogev-Seligmann et al., 2012). A complementary constrained-action perspective predicts that consciously monitoring an automatized skill (an “internal focus”) degrades automaticity, whereas an external focus (e.g., on environmental or task cues) preserves fluid control and frees resources for the secondary task (Wulf and McNevin, 2003).

A useful framework to classify the different behaviors of individuals performing cognitive-motor DT has been proposed by Plummer and Eskes (2015). It relies on the ability to measure quantitative parameters assessing the quality of the motor and the cognitive performances. For any measured parameter X, either belonging to cognitive or motor performance, the dual-task cost (DTC) DX (expressed in %), is defined by Wrightson et al. (2016) as


$$\begin{array}{l} \text{DX}=100\frac{{\text{X}}_{DT}-{\text{X}}_{ST}}{{\text{X}}_{ST}}, \end{array}$$ (1)

with X measured in dual task (*DT*) or single task (*ST*) settings. Within this framework, the different DT patterns are represented by the values, especially the signs, of two DTC: DX cognitive and DX motor. Some individuals will prioritize walking at the expense of cognitive tasks (Gait Priority Trade-Off, GPTO), while others will prioritize cognitive tasks, thereby negatively impacting their motor control (Cognitive Priority Trade-Off, CPTO). In some cases, DT leads to a joint deterioration in motor and cognitive performances (Mutual Interference, MI), while in other situations it can simultaneously improve both aspects (Mutual Facilitation, MF) (Plummer and Eskes, 2015).

Our research question was whether individual experiential characteristics and baseline capacities may be related to DT cognitive-motor performance. Rather than seeking to establish causal determinants, the present exploratory study aimed to characterize inter-individual cognitive-motor profiles by integrating quantitative gait and cognitive measures with perceived stress and explicitation-interview data. A secondary aim was to assess the potential value of this mixed-method approach for future clinical and rehabilitation research.

As the cognitive task, we used the Raven's Progressive Matrices Test (RPMT), a widely used, language-reduced measure of non-verbal reasoning / fluid intelligence (Raven and Kratzmeier, 2003; Raven et al., 2003). The RPMT comprises 60 items, grouped in five sets of 12 items, with increasing difficulty within a set and set by set. Each item presents a 3 × 3 matrix of images, with one image missing. To solve an item, one has to identify the underlying pattern linking one image to another (rotation, addition/subtraction of elements, etc.) and choose the correct image that completes the matrix from a set of options. Combined with treadmill walking, this DT setup provides a controlled environment for assessing cognitive and motor interactions. As the treadmill is instrumented, the measurement of ground forces and center of pressure allows for the extraction of quantitative parameters describing gait. We focused on gait variability that has already proven to be significantly modulated by DT and RPMT in particular (Dierick et al., 2020). RPMT itemized format also permits trial-level response times and accuracy, which we summarize as cognitive DT costs (DRS) to align with the gait metrics.

Participants. individual experiential states were analyzed using an explicitation interview (EI) after the DT condition. An EI (Guigue-Durning, 1996) makes possible to refine and deepen each layer of lived experience – cognitive, emotional, sensory, and ego-related – established by an individual within a specific and singular context during the performance of various actions, both in their explicit (reflective) and implicit (pre-reflective) dimensions. Guiding the EI requires particular expertise (and therefore training) but also sustained attention, constant reflexivity, and the ability to mentally share one's own subjective experience with that of the person conducting the interview. The exploration of layers of lived experience is only possible once the learner is reconnected with their past experience. It is therefore necessary to guide them toward an “embodied position of speech”, or a posture of inner availability, to access the gradual evocation of this experience, as it was lived at the time it was lived. As an EI unfolds, the participants gradually carve out a path toward the description of this past experience. The knowledge underlying the entirety of lived action and the goals pursued by the individual also helps to shed light on the logic of action in which the subject is engaged. Indeed, various “satellites” continuously orbit around a lived action: The context, which allows the individual to reconnect with their specified experience; Experiential knowledge (gained through experience) and theoretical knowledge (prescribed) that guide singular actions within this specified context; The constant and operative subjective evaluations present during the task (e.g., beliefs, values, and opinions). The intentions and aims toward which the specified action is directed. The EI is particularly well suited to this research question because it is specifically designed to access the pre-reflective dimensions of lived experience that operated during the actual action, rather than relying solely on participants' *post-hoc* explanations or interpretations. It therefore provides fine-grained access to the cognitive, attentional, emotional, and bodily processes involved in managing the DT situation, offering a unique qualitative complement to behavioral performance measures and helping to illuminate the experiential processes underlying the different performance profiles. EI was therefore the most appropriate method for accessing the participants' proprioceptive issues, as well as the cognitive mechanisms involved in decision-making regarding the RPMT task.

We propose a model (Figure 1) linking three determinant sets to DT outcomes: (1) baseline capacities: gait automaticity/variability at ST and cognitive ability (e.g., RPMT); (2) situational affect: eustress vs. distress (brief perceived stress and EI affective tone); and (3) control policy: strategy focus (analytical/monitoring vs. automatized/external) and sensory immersion (engagement with external auditory/visual cues vs. self-referential rumination), both inferred from EI. These determinants influence attentional allocation and motor automaticity, yielding one of four outcomes: MI, GPTO, CPTO, or MF.

**Figure 1:**
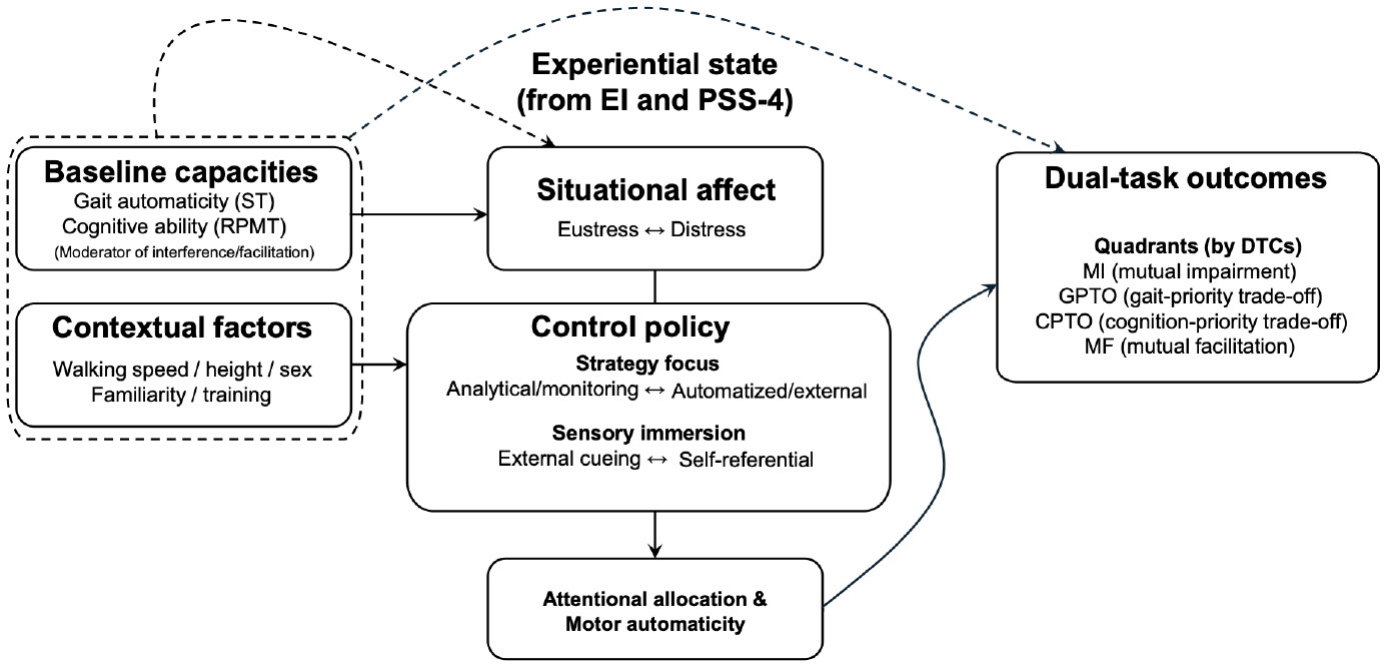
Conceptual model of experiential control in dual-task gait. Baseline capacities and contextual factors combine with the experiential state [derived from explicitation interviews and a brief perceived-stress index (PSS-4)], comprising situational affect and control policy (strategy focus and sensory immersion), to shape attentional allocation and motor automaticity. This allocation yields four outcome profiles defined by cognitive and motor dual-task costs (DTCs): mutual impairment (MI), gait-priority trade-off (GPTO), cognition-priority trade-off (CPTO), and mutual facilitation (MF).

The aim of this study was to explain inter-individual differences in cognitive–motor trade-offs during DT walking by integrating EI-derived experiential states with quantitative DTC. Given the exploratory nature of the study, four a priori theoretical expectations (E) were used to guide integration and interpretation of the quantitative and qualitative findings. These expectations constitute a conceptual framework for exploratory interpretation rather than predictions tested statistically in the present sample.

**E1—Distress and prioritization**. Greater distress was expected to co-occur with less efficient cognitive-motor management, including patterns characterized by cognitive deterioration and/or motor interference.

**E2—Strategy and engagement**. Distracting thoughts, reduced engagement with the cognitive task, or increased internal monitoring were expected to co-occur with less favorable cognitive-motor patterns than more automatized or externally focused strategies.

**E3—Eustress and engagement**. Positive or manageable challenge appraisal, cognitive engagement, and sensory immersion were expected to occur more frequently in profiles characterized by preserved/facilitated cognitive performance or mutual facilitation than in profiles characterized by marked interference.

**E4—Baseline capacity**. Higher baseline cognitive performance and gait automaticity were expected to be associated descriptively with greater preservation of performance across conditions.

## 2 Materials and methods

### 2.1 Participants

Young healthy adults were recruited for this study; inclusion and exclusion criteria are given in Table 1. Participants were required to abstain from alcohol and narcotics for 24 h before measurement sessions. Participants were also asked not to have undergone any training on the RPMT within the 6 months preceding the study or between the two evaluation sessions. Indeed, it has been demonstrated that a single training session significantly improved performance across all age groups (Denney and Heidrich, 1990).

**Table 1:** Inclusion and exclusion criteria of our study.

Inclusion	Exclusion
To be between 18 and 25 years old.	To have trained on Raven's progressive matrices in the 6-month interval between the two measurements or to remember items.
To be enrolled at HELHa in Montignies-sur-Sambre.	To have a diagnosed dys disorder, concentration disorder or cognitive disorder diagnosed by a professional.
To be free from medication and any neuromusculoskeletal, orthopedic, respiratory, or cardiovascular disorders that could affect gait.	
To be able to walk on a treadmill without aid.	
Not to have consumed drugs, medication, or alcohol in the 24 h prior to the measurement sessions.	

Data collection occurred in two phases: February-March 2024 (*T*_1_) and October 2024 (*T*_2_). Ethics approval for the study was obtained from the Brussels Alliance for Research and Higher Education Academic Ethical Committee (Protocol Number: B200-2024-054). All participants provided written informed consent.

No a priori power calculation was performed. Recruitment yielded a convenience sample from a single institution, and the sample was not prospectively sized to detect a pre-specified effect or to support multivariable prediction of profile membership.

### 2.2 Protocol

Informed consent, age, sex, body mass, height, and physical activity (hours of sport per week) were collected for all participants at *T*_1_. During this session, the comfortable walking speed, *v*, was established during a familiarization session on the treadmill for each participant, following the protocol described in Dierick et al. (2020). Participants wore comfortable running shoes and were instructed to keep their gaze forward during treadmill walking. Treadmill speed was progressively increased in increments of 0.1 km h^−1^. When the participant indicated that the speed felt too fast, it was decreased by the same increment until a comfortable walking speed was reached. The familiarization period lasted between 1 and 3 min for all participants. The comfort speed was then maintained for all walking trials. After the familiarization period, participants were engaged in a 10-min treadmill walk, during which the vertical component of the ground reaction force (*F*_*v*_) and center of pressure (*CoP*) were recorded at 500 Hz using CueFors 2 software (Motekforce Link, The Netherlands). The same experimenter (FJ) managed the treadmill measurements.

Next, the RPMT (Raven and Kratzmeier, 2003; Raven et al., 2003) was subsequently administered to the participants in a single-task (ST) setting, i.e., seated in a quiet room, completing a paper form, without experimenter (EH) interaction. The experimenter recorded participants' responses and the time taken to answer each item. These measurements led to RPMT Score (RS, /60) and time (T) in ST.

At *T*_2_, participants were first asked to complete the PSS-4 questionnaire (Cohen et al., 1983). The RPMT was then projected onto a screen located in front of the treadmill and administered to the participants in a DT setting: They responded orally while engaged in a second treadmill walk, whose duration was the time needed to complete the 60 items. The same data were collected by the two experimenters in charge of the measurements (EH and FJ) as in *T*_1_. At the end of the DT session, participants were asked if they had randomly answered to at least one item of the RPMT or not. Immediately after the *T*_2_ DT RPMT, participants were asked a single retrospective binary question indicating whether they believed that they had answered at least one RPMT item at random (yes/no). More precisely, the question was “Did you answer to at least one item without having tried to understand the logic behind it?”. Thus, “self-reported random responding” denotes the report of at least one such response and does not quantify the number or location of affected items. Item-level guessing or confidence was not recorded.

Participants subsequently completed an EI in a separate quiet room. All interviews were conducted by the same interviewer (SB), recorded, and subsequently transcribed verbatim. The interview focused retrospectively on the participant's experience during the DT condition, including cognitive strategies, emotional experience, and bodily and attentional sensations. During the interview, the participant and the experimenter conducting the interview sat side by side in a quiet room, avoiding any eye contact that might lead to a search for approval or disrupt the subject's concentration. The interview was recorded so that it could be transcribed verbatim afterwards. The experimenter began by asking for the subject's consent with a simple sentence: “If you agree, I suggest we go back to the moment when you entered the lab. Take your time and when you are ready, give me a sign.” The purpose of this verbal prompt is to anchor the participant in a revival of the entire subjective experience related to the specific moment when they entered the laboratory—which will later be referred to as the pre-start of the task − as well as the cognitive strategy adopted by the subject during the test, their feelings during the test and their internal sensations. By exploring the participant's thoughts and behaviors, the EI highlights the invisible aspects of their subjective experience of a specific action, in this case the DT (Denis and Winkopp, 2022; Petitmengin et al., 2015).

### 2.3 Data analysis – dual-task costs

The RPMT score, RS, and time to complete, T, were recorded for all participants in ST and DT. From *F*_*v*_ and *CoP* time series recorded for all participants in ST and DT, the time series containing the durations of the successive gait cycles were computed using CueFors2 software, a gait cycle being defined as the time interval between two consecutive heel strikes of the left foot. Typical traces of gait cycle durations are shown in Figure 2.

**Figure 2:**
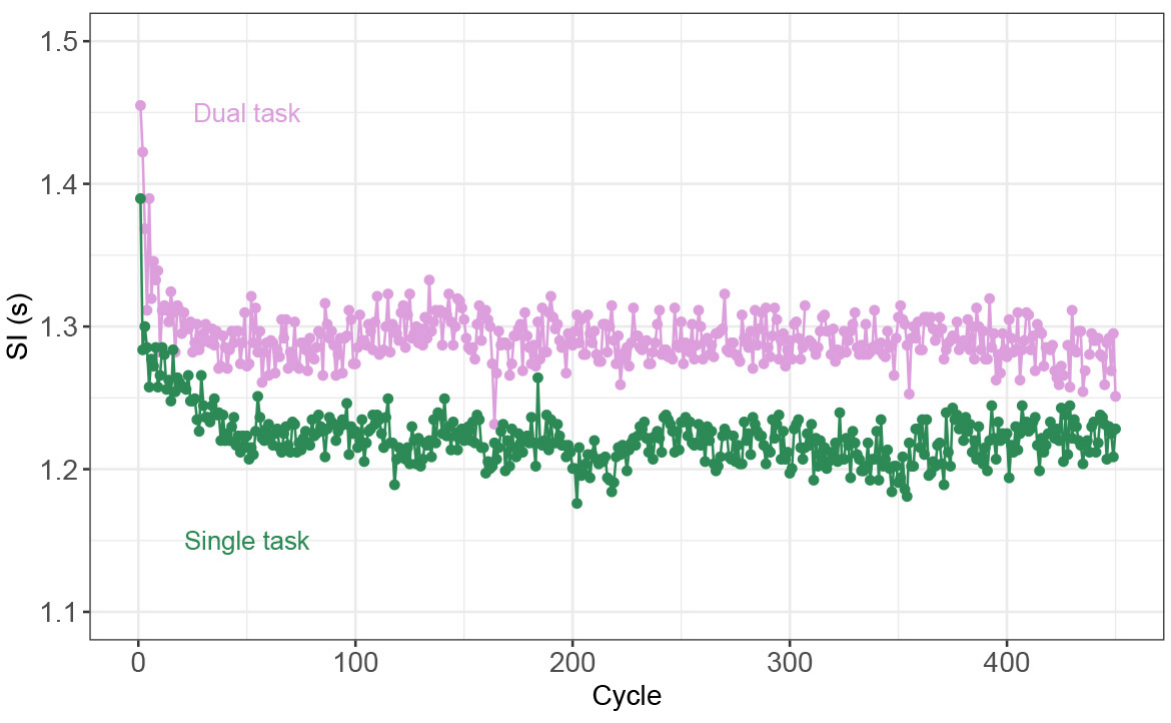
Typical traces of gait cycle duration time series in single task (green) and dual task (purple).

From the latter time series, the following parameters were computed: the average duration of gait cycles, i.e., the stride interval SI, the coefficient of variation, CV, the Hurst exponent, H, and the fractal dimension, D. CV is the ratio of gait cycle duration standard deviation to SI; it is a measure of the magnitude of gait variability. H has been computed using detrended fluctuation analysis (DFA) according to the method presented in Phinyomark et al. (2020); Dierick et al. (2017). H is a measure of the predictability of the time series: H close to 0.5 is characteristic of a random process, while intermediate values between 0.5 and 1, observed in healthy adults, are typical of autocorrelated chaotic systems. D was computed using the box count method (Kantz and Schreiber, 2004; Dierick et al., 2017). D∈[1, 2]. The closer D is to 1, the more the gait cycle durations varies smoothly from one cycle to another. The more D is close to 2, the more the time series are complex, i.e., gait cycle duration changes abruptly from one cycle to another. All these computations were done using home-made routines in R software. Other spatio-temporal or stability measures might also be sensitive to DT effects, but our choice of indices has already shown relevance in both healthy and pathological gait analysis (Dierick et al., 2021; Moon et al., 2016).

Since all parameters were computed both in single and in dual task, the corresponding DTCs were finally computed according to Equation 1. DTCs were then used to match the observed behaviors with the categories defined by Plummer and Eskes (2015). DRS is the simplest index assessing the cognitive modification induced by DT: DRS>0 (resp. < 0) is related to an increase (resp. decrease) in cognitive performance during DT condition. A relevant index assessing motor performance needs to show a monotonic behavior, i.e., larger or smaller values always correspond to increased or decreased motor performance. As in Dierick et al. (2025), we concluded that the best candidate was CV: All studies on gait variability agree that any deviation from the optimal gait pattern leads to a larger CV, see, e.g., the review of Moon et al. (2016). A negative (resp. positive) DCV can always be associated with an increased (resp. decreased) motor performance. The other variability indices X we measured have rather optimal values (Dierick et al., 2021): Any DX≠0 is presumably linked to a decrease in motor performance. These indices cannot be regarded as relevant motor indices regarding Plummer's classification.

DCV < 0 can be straightforwardly related to gait facilitation since it corresponds to a better control of gait cycle duration. We therefore represent individual results as points in a (DRS, DCV) plane. The four cognitive-motor profiles are “gait-priority trade-off (GPTO)” (DRS < 0, DCV < 0), “mutual facilitation (MF)” (DRS>0, DCV < 0), “mutual interference (MI)” (DRS < 0, DCV>0), and “cognitive-priority trade-off (CPTO)” (DRS>0, DCV>0). Because the cognitive component may also be influenced by order, practice, administration modality, and task engagement, the resulting categories are used as exploratory descriptive profiles within the present protocol.

For each participant, the distance $\delta =\sqrt{{\text{DRS}}^{2}+{\text{DCV}}^{2}}$ was finally computed. The larger δ, the stronger the impact of DT on a participant.

### 2.4 Data analysis – explicitation interviews

The EIs were analyzed in detail using a data processing methodology developed by Denis (2016). To do this, we must keep in mind that the capture of one's lived experience is precisely situated in space and time (Petitmengin, 2006). The temporal organization of the lived experience was the first step to explore the data. The first reading of the verbatim transcript does not immediately give a logical structure of the different actions carried out by the participants. The gathering of the various processes experienced is thus achieved through a “chronotext” written in “I” (Théron, 2014; Denis, 2016). The chronotext takes up the temporal and sequential structure of the participants' lived experience. Each action (procedural, cognitive, inner gesture, and emotion) is reordered and repeated line by line. To do this, only the action verbs and the information linked to these actions were included. Then, the sentences included were grouped into four dimensions given in Table 2. The four dimensions provided a descriptive framework for organizing the experiential material elicited during the EI. The egoic dimension refers to the role participants assign themselves in the remembered situation. It has been divided into four descriptive categories: The participants could think that they were competing against themselves (self vs. self), against others (self vs. others), or both (self vs. self vs. others). A final category is the absence of competition feeling (none). The cognitive dimension refers to mental operations such as decision-making, problem-solving, reasoning, and planning. It was also divided into four descriptive categories: no strategic thinking, meaning that the participant did not focus on the cognitive part of the experiment, strategic thinking, i.e., they developed a specific cognitive strategy to respond to the different RPMT items, distracting thoughts, i.e., thoughts that were unrelated to the cognitive task at hand. Finally, there were participants who experienced both distracting and strategic thoughts. The emotional dimension identifies the nature of the emotions felt, their intensity, their origin, and their role in the dynamics of the action. Five emotional categories were identified from the verbatim transcripts: sadness, fear, shame, joy, and anger (Ekman and Friesen, 1971). It is important to note that the examiner did not necessarily record the emotion expressed directly by the participant, but rather a personal and retrospective interpretation based on the comments provided. Finally, the physical dimension encompasses posture, muscle tension, internal sensations, and spontaneous gestures. The body is understood here as the embodied memory of action. Evoking physical sensations can serve as a gateway to other dimensions (Maurel, 2009). We divided this dimension into four categories. First, the participant was in “automatic gait” mode, not concentrating on walking. The “hearing” category contains participants who could hear what was going on around them and were therefore not solely focused on the cognitive task. Participants could also be classified as belonging to two other categories, namely, “automatic gait + hearing?”, which means that they were in the first two items simultaneously, or the “no” category, which means that they were not aware of either of the first two items.

**Table 2:** Four dimensions selected to analyse the parts of the explicitation interviews related to the dual-task experience, and the different cases representative of participants' experiences in the different dimensions.

Egoic	Physical	Cognitive	Emotional
Self vs. self and others	Automatic gait + hearing	Strategic thinking + distracting thoughts	Anger
Self vs. self	Automatic gait	Strategic thinking	Fear
Self vs. others	Hearing	Distracting thoughts	Sadness
None	None	None	Shame
			Happiness

The qualitative material was coded independently by two researchers (SB and JD) using an independent double coding. Following independent coding, SB and JD compared their respective coding decisions. Any discrepancies were discussed between the two researchers and resolved through consensus. This procedure was intended to ensure that the final categorization resulted from an explicit comparison of the researchers' interpretations rather than from the unilateral interpretation of a single researcher. The same independent double coding procedure was used for the selection of verbatim excerpts presented in the Results section. SB and JD independently selected excerpts considered representative and illustrative of the coded experiential dimensions without access to the participants' quantitative DT profiles. The selected excerpts were subsequently compared and discussed until consensus was reached. The coding remained primarily descriptive and closely anchored to participants' accounts. It was not intended to generate an exhaustive thematic taxonomy or to estimate the prevalence of experiential phenomena. Rather, the purpose was to structure the lived-experience data and to facilitate their subsequent comparison with the quantitative DT profiles. Importantly, the coding was conducted independently of the quantitative classification, which was only considered at the subsequent stage of integration of the qualitative and quantitative findings. In summary, the analytical procedure consisted of seven successive steps given in Table 3.

**Table 3:** Summary of the seven steps followed to analyse the explicitation interviews.

Step	Category	Procedure
1	Transcription	Verbatim transcription of all explicitation interviews.
2	Chronotext	Chronological reconstruction of the lived experience in the first person, retaining procedural actions, cognitive operations, inner gestures, and emotional experiences.
3	Categorization	Organization of the material into four dimensions: egoic, cognitive, emotional, and physical.
4	Double coding	Independent coding by SB and JD, both blinded to participants' quantitative dual-task profiles.
5	Consensus	Comparison of coding and discussion of discrepancies until consensus was reached.
6	Verbatim selection	Independent selection of illustrative excerpts by SB and JD, followed by comparison and consensus.
7	Mixed-methods integration	Qualitative findings subsequently interpreted in relation to the quantitative dual-task profiles.

### 2.5 Statistical analysis and mixed-methods integration

Descriptive statistics were first used to characterize the sample and the distributions of the DTC outcomes. For each of the eight DTC measures (DRS, DT, DSI, DCV, DH, DD, DDS, and Dw), the difference from zero was examined using a two-sided one-sample t-test when the distribution was considered compatible with normality and a two-sided Wilcoxon signed-rank test otherwise. Effect sizes and 95% confidence intervals were reported alongside *p*-values. Cohen's *d* was used for one-sample t-tests and matched-pairs rank-biserial correlation *r* for Wilcoxon signed-rank tests.

In addition, a t-test was used to compare DTC between participants who reported responding randomly or not. In cases of non-normality of the data, these comparisons were performed using the Mann–Whitney test. Hedges' *g* was used for parametric comparisons and rank-biserial correlation *r* for Mann–Whitney comparisons. Ninety-five percent confidence intervals were calculated analytically for means and mean differences and using percentile bootstrap resampling for non-parametric estimates and effect sizes.

To address multiplicity, Holm's sequential correction was applied separately to two families of analyses: (1) the eight tests comparing DTC outcomes with zero and (2) the eight comparisons of DTC outcomes according to self-reported random responding. Both unadjusted and Holm-adjusted *p*-values are reported.

Participants were also classified descriptively according to their position in the (DRS, DCV) plane. Given the small number of participants within each profile, no multivariable model of profile membership was performed. DTC and EI were analyzed independently before integration. In particular, the qualitative coding and selection of illustrative verbatims were conducted by SB and JD while both researchers were blinded to participants' quantitative profiles. The DTC calculation was carried out by the other authors and was therefore not used to define, modify, or guide the qualitative coding. Integration occurred subsequently at the interpretive stage. The experiential characteristics identified through the EI were considered in relation to the four quantitative profiles to identify convergent or contrasting experiential configurations. The two participants with the largest δ within each profile were examined in detail to illustrate pronounced individual patterns. This integration was therefore case-oriented and interpretive rather than statistical. Given the exploratory design, the small number of participants within each profile, and the purposive selection of extreme cases, the qualitative findings were not interpreted as evidence of profile-level associations. Instead, they were used to characterize possible experiential and strategic configurations and to generate hypotheses for future research.

## 3 Results

### 3.1 Population

Forty-four participants were assessed at *T*_1_. Ten did not return for *T*_2_, and one participant was excluded at *T*_2_ because alcohol had been consumed within the preceding 24 h. The final analytical sample therefore comprised 33 participants who completed both sessions. Table 4 summarizes the characteristics of the 33 participants included in the study.

**Table 4:** General characteristics of participants at *T*_1_.

N	33
Gender (M/F)	21/12
Mass (kg)	75.2 ± 12.2
Height (cm)	174 ± 11
*v* (km h^−1^)	3.8 [3.7, 4.1]
Sport (h week^−1^)	2 [1, 3]
RS_*ST*_ (/60)	46 [42, 50]
CV_*ST*_	0.0212 [0.0169–0.0295]
H_*ST*_	0.747 [0.695–0.790]

Data are given either under the form mean ± standard deviation or median [Q1, Q3]. The values of RS, CV, and H are given in ST.

### 3.2 Dual-task costs and categories

The eight DTC outcomes are graphically displayed under the form of violin plots in Figure 3. They are also summarized in Table 5 together with effect sizes, 95% confidence intervals, unadjusted *p*-values, and Holm-adjusted *p*-values. After correction for multiple testing, only RPMT completion time (DT) differed significantly from zero. Median DT was −20.60%, corresponding to a large matched-pairs rank-biserial effect (*r* = −0.78; unadjusted *p* < 0.001; Holm-adjusted *p*= 0.0009). None of the remaining cognitive or gait DTCs differed significantly from zero after Holm correction.

**Figure 3:**
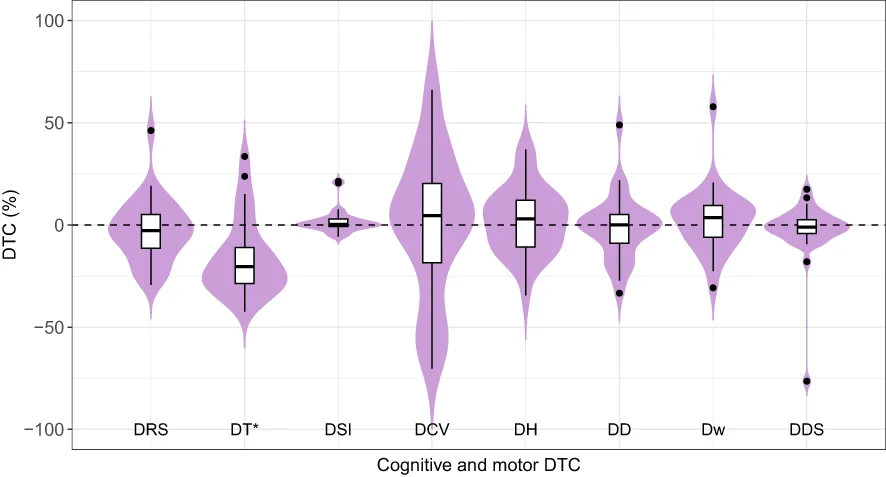
Violin plots of the computed DTC. A * indicates a significantly non-zero DTC regarding Holm-adjusted *p*-values. The dashed line shows the line DTC = 0.

**Table 5:** Dual-task costs of the measured parameters.

DTC (%)	Estimate	Effect size [95% CI]	Unadjusted *p*	Holm-adjusted *p*
DRS	−2.86 [−11.36, 5.13]	*r* = −0.27 [−0.63, 0.13]	0.191	1.000
DT	−20.60 [−30.47, −11.40]	*r* = −0.78 [−0.97, −0.52]	**< 0.001**	**0.0009**
DSI	0.30 [−0.78, 2.89]	*r* = 0.22 [−0.16, 0.59]	0.268	1.000
DCV	6.11 [−16.29, 26.89]	*r* = 0.16 [−0.24, 0.54]	0.442	1.000
DH	2.25 ± 16.15	*d* = 0.14 [−0.21, 0.49]	0.429	1.000
DD	−1.33 ± 15.53	*d* = −0.09 [−0.48, 0.24]	0.627	1.000
DDS	−1.03 [−4.26, 2.55]	*r* = −0.14 [−0.53, 0.27]	0.483	1.000
Dw	3.54 [−6.03, 9.52]	*r* = 0.15 [−0.24, 0.53]	0.469	1.000

Data are given either as mean ± standard deviation or median [Q1, Q3]. Effect sizes are reported as Cohen's *d* for parametric one-sample comparisons and matched-pairs rank-biserial correlation (*r*) for non-parametric comparisons, with 95% confidence intervals. Raw and Holm-adjusted *p*-values are reported. Significant *p*-values are written in bond font.

Twenty-five participants reported having answered at least one RPMT item randomly during the *T*_2_ session, whereas eight reported no random responding. Participants reporting random responding showed lower DRS than those reporting none (median −4.44 vs. +6.92%; *r* = −0.55; unadjusted *p* = 0.022) and higher DH (5.71 vs. −8.55%; *g* = 0.92; unadjusted *p* = 0.042). However, neither comparison remained statistically significant after Holm correction across the eight DTC group comparisons (DRS: Holm-adjusted *p*= 0.176; DH: Holm-adjusted *p*= 0.297). Detailed data are presented in Table 6.

**Table 6:** Comparison of dual-task costs between participants who self-reported at least one random response during the RPMT (Yes, *n* = 25) and participants who reported no random responses (No, *n* = 8).

**DTC (%)**	Random Yes	Random No	Effect size [95% CI]	Unadjusted *p*	Holm-adjusted *p*
DRS	−4.44 [−16.67, 2.04]	6.92 [−2.66, 11.07]	*r* = −0.55 [−0.86, −0.18]	**0.022**	0.176
DT	−25.19 [−31.83, −12.62]	−15.73 [−20.32, 10.39]	*r* = −0.41 [−0.80, 0.06]	0.089	0.533
DSI	−0.18 [−1.71, 2.96]	0.58 [0.17, 1.67]	*r* = −0.21 [−0.57, 0.18]	0.389	1.000
DCV	7.14 [−24.97, 26.89]	2.86 [−1.40, 12.96]	*r* = −0.05 [−0.45, 0.37]	0.850	1.000
DH	5.71 ± 15.04	−8.55 ± 15.50	*g* = 0.92 [0.22, 1.78]	**0.042**	0.297
DD	0.00 [−7.02, 3.18]	0.01 [−18.11, 8.21]	*r* = 0.04 [−0.48, 0.55]	0.883	1.000
DDS	−1.03 [−4.26, 2.55]	−2.22 [−4.70, 1.96]	*r* = 0.07 [−0.37, 0.51]	0.785	1.000
Dw	2.47 [−6.72, 6.74]	9.43 [−4.54, 10.68]	*r* = −0.20 [−0.67, 0.29]	0.413	1.000

Data are given either as mean ± standard deviation or median [Q1, Q3]. Effect sizes are reported as Hedges' *g* for parametric comparisons and rank-biserial correlation (*r*) for non-parametric comparisons, with 95% confidence intervals. Raw and Holm-adjusted *p*-values are reported. Significant *p*-values are written in bold font.

Within our sample, the distribution of participants according to the classification of Plummer and Eskes (2015) showed that all four profiles were represented as shown in Figure 4. There are 12 participants in the MI category, nine in GPTO, seven in CPTO and five in MF. To illustrate pronounced expressions of each profile, the two participants with the largest distance δ within each category were selected for detailed qualitative examination. These participants are considered illustrative extreme-profile cases and should not be interpreted as statistically representative of all participants assigned to the corresponding profile. We identified participants 8 (δ = 40.3) and 29 (δ = 47.0) for the MI category; participants 11 (δ = 54.1) and 27 (δ = 59.9) for GPTO; participants 24 (δ = 66.2) and 33 (δ = 68.6) for CPTO; participants 21 (δ = 19.5) and 22 (δ = 29.5) for MF. It is important to note that participant 1 (δ = 75.1), although extremely marked in the GPTO category, was excluded in view of further analysis. This is because their EI was conducted by one of the authors (JD), expert in the field, to train the experimenter who conducted all the other interviews (SB). To ensure methodological consistency, participant 1 was not included in the comparative analysis of the profiles. The DTCs of the identified participants are given in Table 7.

**Figure 4:**
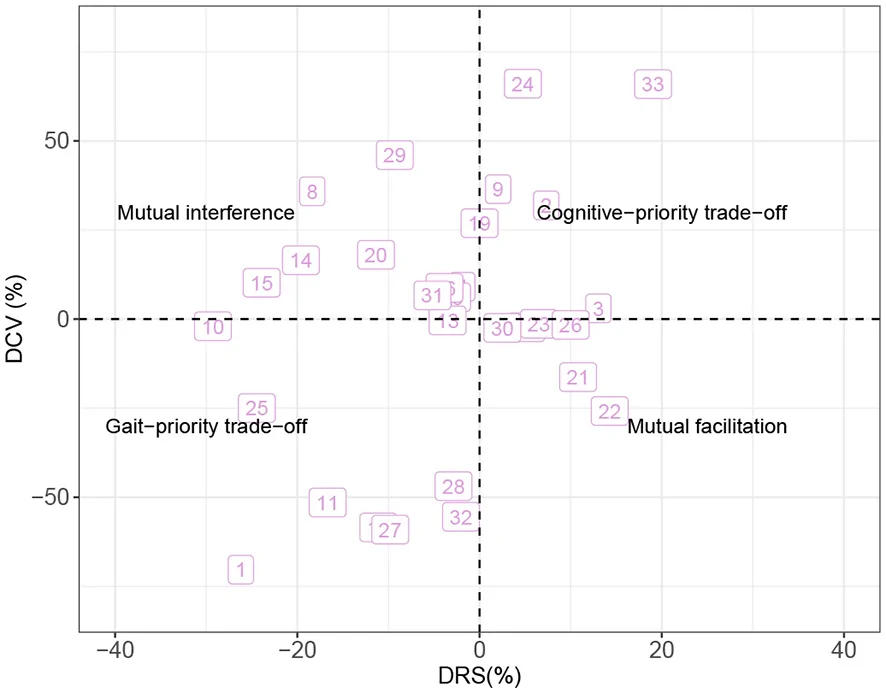
Distribution of participants across the four categories identified by Plummer and Eskes (2015). Cognitive performance is measured by DRS, and motor performance is measured by DCV. The numbers label participants.

**Table 7:** Dual-task costs (in %) of the two participants with the largest δ in each category.

**Case**	CPTO		GPTO		MF		MI	
Participant	24	33	11	27	21	22	8	29
DT	−27.7	−20.1	−28.1	0	−12.6	−20.2	−27.2	−1.52
DRS	4.71	19.0	−16.7	−9.80	10.8	14.3	−18.4	−9.30
DSI	−1.10	1.77	−2.62	−5.45	21.3	0.304	−4.26	−3.82
DCV	66.1	65.9	−51.5	−59.1	−16.3	−25.8	35.9	46.0
DH	−10.75	−12.8	−6.58	37.0	7.62	−11.3	3.06	18.1
DD	−21.9	−19.6	3.08	19.1	1.24	7.92	0	−2.25
DDS	–1.15	4.71	2.00	−18.0	13.3	−9.31	−7.92	−2.59
Dw	5.15	−6.03	4.90	2.47	−5.73	13.5	−14.0	−17.6

### 3.3 Explicitation interviews

The radar plots of the two participants with the largest δ for each category defined by Plummer and Eskes (2015) are displayed in a single Figure 5 to appraise the differences between the participants' profiles. Relevant quotations of their EI are given in Tables 8, 9. We refer the interested readers to the OSF repository *https://osf.io/jpbdc/*, where all the radar plots and verbatims of explicitation interviews are available.

**Figure 5:**
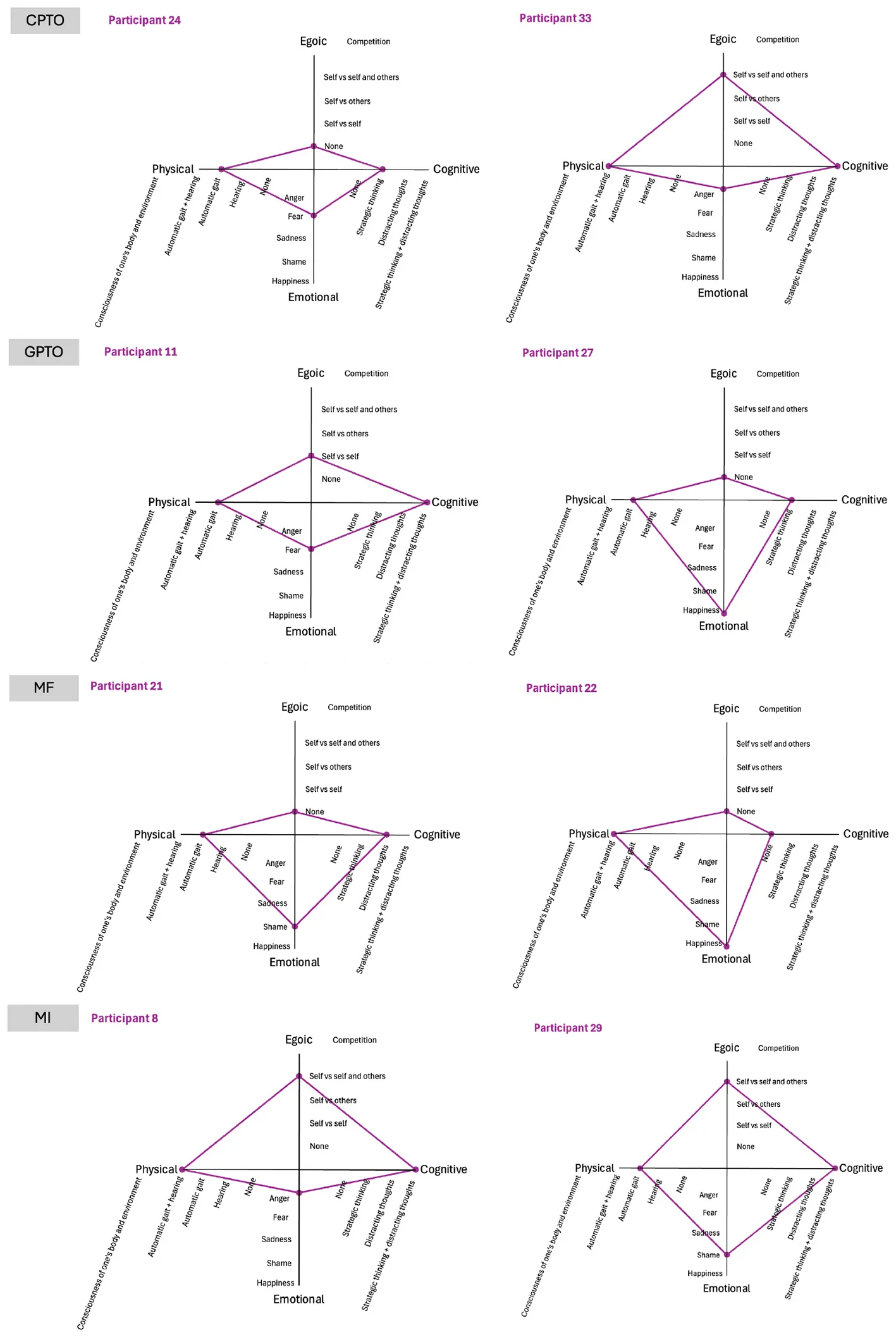
Radar plots summarizing the explicitation interviews of the two most representative participants, i.e., participants with the largest distances δ, in the four categories of Plummer and Eskes (2015). Selected verbatims of those participants are given in Tables 8, 9.

**Table 8:** Selected verbatims from the explicitation interviews of the two most representative participants, i.e., participants with the largest distances δ, in the categories CPTO and GPTO of Plummer and Eskes (2015).

	CPTO	
Dimension	Participant 33	Participant 24
Egoic	Me against myself. Doing better than others. Well, than me, anyway. And then, above all, others, I think, actually.	No, not at all [stressed].
Physical	Yes, I heard [the treadmill or my footsteps] at first. And after that, I didn't pay any more attention.	All I could see was the white board. My peripheral vision was practically non-existent. But the periphery doesn't matter anymore. It's really what I'm doing that matters. I play basketball, so when it comes to free throws, even if the opposing crowd is yelling anything at you, I'm in my own world and it doesn't bother me.
Emotional	PSS-4: 7	PSS-4: 6
	I wasn't stressed at all. I told myself that it was over. I wanted to do better than the first time. I was a bit competitive.	The first time, yeah. I don't know why, even though I wasn't walking. But then, walking, I don't know, it felt like a little stroll, a bit of reflection anyway. But now, I preferred that. I really wasn't stressed.
Cognitive	I was trying to see if there was any logic to these letters. Then I started to... I looked at the first line of the and I was trying to... At right angles, actually. With the first horizontal line and the first vertical line.	I didn't remember, but I still knew exactly what we were going to do, so I felt comfortable, more comfortable than last time. Oh yes, it was the exercises [that I prioritized]. Because the speed of the treadmill was almost like my usual walking pace. So it felt natural.
	**GPTO**	
Dimension	Participant 11	Participant 27
Egoic	I was a bit stressed about my performance, I think. Am I bad at this, or... I often tell myself that.	Ah, no, not at all [stressed].
Physical	I was completely absorbed in the screen. I didn't even realize I was walking.	[I didn't] hear a teacher come in at all. No, it was automatic [the walk].
Emotional	PSS-4: 9	PSS-4: 5
	A bit of both [stressed about doing badly, taking too long]. In my mind, tests are a mix of going fast and doing well, so I felt a bit of pressure. I was focused, but at the same time I was a bit stressed.	No, not at all [no pressure].
Cognitive	When it was a nine-figure exercise, for example, I would try to see the similarities vertically and horizontally in my rows to try to find the missing logic.	I look at the screen, I don't pay attention to anything else. I focus.

The quotations are grouped by dimensions of the radar plots (Figure 5). The PSS-4 score is given in the emotional part.

**Table 9:** Selected verbatims from the explicitation interviews of the two most representative participants, i.e., participants with the largest δ, in the categories MI and MF of (Plummer and Eskes 2015).

	**MF**	
Dimension	Participant 21	Participant 22
Egoic	I'm in a laboratory that I know, with people I know, so... No more worries than that. No competition, it's just... to be a little serious anyway.	No, not at all [stress]. No [pressure to score].
Physical	I didn't pay any attention to it [the noise of the treadmill]. [I was focused] only on the exercise, nothing else.	Finally, I can't hear anything anymore, in quotation marks [neither the treadmill nor my footsteps].
Emotional	PSS-4: 5	PSS-4: 7
	If I had slept a little longer, I could have managed.	
Cognitive	I constantly ask myself, “Could I have done better?”	I just looked at the shapes each time. The piece that fit best, let's say. And then, well, I validated it, or rather, I checked it and then validated it.
	**MI**	
Dimension	Participant 8	Participant 29
Egoic	I told myself I would do my best and then... And then that was it. I think it was to be within the norms.	I attach a lot of importance to how others see me. So, it sounds silly, but I tell myself every time that if I make a mistake, since they have the answers, it would look stupid.
Physical	To be honest, I didn't even think about walking.	I heard someone come in behind me for a moment. [The treadmill] didn't bother me, but I was also relying on it a little, as if it gave me a rhythm. [I felt] as if I were inside the screen.
Emotional	PSS-4: 9	PSS-4: 12
	I wasn't stressed, but it annoyed me that I couldn't always see the logic behind items. It frustrated me.	
Cognitive	It's true that there were a few times when I really couldn't see the logic.	I said to myself “OK, isn't there a vertical logic?” So I looked vertically, I saw nothing. Then there was nothing horizontally. Then there was nothing diagonally. Then there was nothing. I looked at the options. I tried to find the ones with the most similar patterns.

The quotations are grouped by dimensions of the radar plots (Figure 5). The PSS-4 score is given in the emotional part.

## 4 Discussion

This exploratory mixed-method study combined quantitative assessment of relative changes in gait and cognitive performance with EI to characterize inter-individual cognitive-motor DT patterns in healthy young adults. The experiential analyses provide descriptive examples of how distress, engagement, strategy, and sensory experience coexist with pronounced DT patterns. We now analyse our four expectations to the light of these analyses.

### 4.1 E1—distress and prioritization

According to E1, greater distress is expected to lead to a posture-first allocation strategy, with preserved or even reduced cognitive performance, thereby increasing the likelihood of a GPTO profile compared with CPTO or MF.

Our results show that distress may modulate DT behavior, but not in a simple “posture-first” fashion as often proposed in postural prioritization models (Woollacott and Shumway-Cook, 1990; Yogev-Seligmann et al., 2012). Participants with the highest perceived stress scores (PSS-4) and verbatim indications of cognitive overload were typically classified in the MI category. They reported strong performance concerns, frustration when failing to identify the RPMT logic, and sensitivity to social evaluation, consistent with a distress appraisal rather than a challenge/eustress appraisal (Le Fevre et al., 2003). Their gait pattern combined increased variability (DCV> 0) with shorter and less adaptive stride dynamics (DSI < 0, DD < 0), consistent with a cautious yet poorly controlled gait under mental overload, while cognitive performance also deteriorated (negative DRS). In these individuals, distress was associated with concurrent deterioration of motor and cognitive performance rather than a clear prioritization of posture, in line with the idea that excessive load can saturate shared resources in DT paradigms (Wickens, 2008; Esmaeili Bijarsari, 2021).

In contrast, GPTO participants systematically adopted a more adaptable gait (DD> 0), indicating that walking became the primary focus during DT. However, distress was not uniformly high in this group: One GPTO participant described negative stress and performance pressure, whereas another experienced the task as relatively relaxed and low stakes. In both cases, cognitive performance declined and participants partly disengaged from the RPMT: We hypothesize that this occurred either as a defensive “refuge” into walking under distress, or as a comfortable, low-pressure choice. This echoes the proposal that prioritization policies are not fixed but emerge from the interaction between perceived task demands, personal goals, and emotional appraisal (Yogev-Seligmann et al., 2012; Le Fevre et al., 2003). Only nine of 33 participants fell into the GPTO category, and treadmill familiarity was high in this student sample, which likely reduced perceived fall risk compared to older adults or neurological populations where posture-first strategies are more consistently observed (Moon et al., 2016; Fritz et al., 2015; Chiaramonte et al., 2022).

Overall, the qualitative observations were partially consistent with E1: Distress and performance concerns were prominent in some participants showing pronounced interference, but the present data do not establish a systematic association between distress and a specific profile.

### 4.2 E2—strategy and engagement

E2 posits that distracting thoughts and/or poor engagement in the cognitive task will lead to greater motor costs and higher stride-to-stride variability compared with an automatized or external focus, thereby increasing the likelihood of GPTO or MI profiles relative to CPTO or MF.

The principal group-level quantitative finding was a shorter RPMT completion time at *T*_2_, which remained statistically significant after correction for multiple testing. In our protocol, task condition was confounded with session order, repeated RPMT exposure, and administration modality. One plausible interpretation is a global reduction in engagement with the RPMT under DT or a practice-related acceleration of reasoning strategies as participants discover solution rules (Verguts and De Boeck, 2002). When participants self-reported having given random answers on at least one RPMT item, this was accompanied by globally worse cognitive performance (DRS < 0) but more stereotyped gait (DH> 0), that is, a more stereotyped stride-to-stride structure. Conversely, participants who denied random responding globally showed improved or preserved RPMT scores (DRS> 0) but a decrease in gait predictability (DH < 0), indicating a more disrupted locomotor pattern. Note that the magnitude of differences between participants having randomly answered or not may be meaningful, but neither remained statistically significant after Holm correction. More importantly, the present binary self-report cannot establish why random responding occurred. It may reflect isolated guessing on difficult items, cognitive overload, reduced engagement, strategic disengagement, or partial task abandonment.

These findings partially contradict the strict formulation of E2 based on the constrained-action perspective, which predicts that consciously monitoring an automatized skill or withdrawing attention from external cues should degrade automaticity and increase motor costs (Wulf and McNevin, 2003). Still, the constrained-action framework remains useful for interpreting individual profiles. MI participants, who experienced high distress and strong self-monitoring of performance, exhibited a gait pattern that was both more variable and less adaptive, in line with the idea that excessive internal monitoring can disrupt automatic motor control (Dierick et al., 2020). In contrast, MF and some CPTO participants appeared to rely on automatized gait or external focus strategies, allowing them to maintain or even improve cognitive performance without destabilizing locomotion.

### 4.3 E3—eustress and engagement

E3 posits that eustress, together with strong engagement and sensory immersion in the cognitive task, will favor MF or CPTO profiles over GPTO or MI, provided that baseline gait automaticity is sufficient.

MF participants reported a positive or neutral emotional tone, low pressure, and a sense of being “immersed” in the RPMT without being overwhelmed. They paid little attention to treadmill noise or external stimuli and described an efficient, sometimes playful engagement with the matrices, consistent with a challenge/eustress appraisal. Quantitatively, they showed improved gait complexity (DD> 0) and preserved or improved cognitive performance, suggesting that their control policy allowed both tasks to benefit from shared resources rather than compete, in line with multiple-resources theory under moderate load (Wickens, 2008).

CPTO participants also demonstrated deliberate cognitive engagement but with a clearer priority given to the RPMT. They adopted either analytical strategies (systematic scanning of rows and columns) or a more automatized approach to the test, while accepting that gait would become less complex and more predictable (DD < 0, DH < 0). Emotionally, some CPTO participants experienced mild stress that was interpreted as motivating, whereas others deliberately downregulated pressure to stay effective, again consistent with eustress rather than distress. This pattern is compatible with the notion that when task demands are perceived as manageable, individuals can flexibly allocate resources and accept controlled degradation of non-prioritized functions (Esmaeili Bijarsari, 2021).

Together, some illustrative MF and CPTO cases described positive or manageable affect, cognitive engagement, and immersion, which is qualitatively consistent with E3. Given the small purposively selected case sample, however, these observations should be regarded as possible experiential configurations rather than profile-level associations.

### 4.4 E4—baseline capacity

E4 posits that higher baseline gait automaticity and cognitive ability will attenuate DT interference and amplify facilitation, thereby strengthening MF profiles and weakening MI and GPTO patterns.

Testing E4 directly is limited by our sample size and design, but some observations are compatible with a capacity-gating interpretation. First, our participants were young, healthy, and physically active, with ST gait complexity and RPMT performance within or above normative ranges. This high baseline capacity may help explain why all four DT profiles could be expressed without overt safety issues, and why even those with MI or GPTO did not show catastrophic gait breakdown, in contrast with some neurological populations where DT can markedly destabilize gait (Fritz et al., 2015).

Second, qualitative data suggest that some CPTO and MF participants were able to flexibly select strategies (analytic or automatized) and to reinterpret mild stress as motivating rather than threatening, which may reflect a relatively large “buffer” of cognitive and motor resources. Conversely, MI participants, despite sharing the same demographic background, may operate closer to their functional limits: High perceived stress and performance concerns seem to saturate their available resources, leading to simultaneous degradation of gait and cognition. However, we did not systematically relate ST gait complexity or RPMT scores to DT profile membership, and our homogeneous sample restricts the range of baseline capacities.

E4 was not formally tested. The homogeneous sample and restricted range of baseline capacities, together with the small profile subgroups, preclude conclusions regarding moderation or capacity gating. The qualitative observations serve only to motivate formal testing in a larger sample.

### 4.5 Methodological limitations and perspectives

Several limitations are central to interpretation of the present findings.

First, the ST and DT sessions were neither counterbalanced nor administered using equivalent procedures. The ST RPMT was always completed at *T*_1_ while seated using a paper-based format, whereas the *T*_2_ RPMT was projected on a screen while participants walked on a treadmill and responses were provided orally. The same RPMT items were therefore encountered twice. Consequently, *T*_1_–*T*_2_ cognitive differences cannot be attributed exclusively to concurrent walking and may also reflect session/order effects, repeated exposure or practice, familiarity with the items, and differences in presentation and response modality. Test/retest improvement exists and is mostly due to learning the strategy to solve RPMT items (Freund and Holling, 2011a,b; Bors and Vigneau, 2003). Participants were asked not to undertake external RPMT training between *T*_1_ and *T*_2_ to avoid learning such strategy. Our results should therefore be interpreted as condition/session contrasts rather than pure estimates of DT interference. Future studies should counterbalance condition order, use equivalent administration modalities, and preferably use validated parallel forms.

Second, self-reported random responding was assessed only retrospectively using a binary yes/no question. The protocol did not record the number or identity of guessed items and therefore cannot distinguish an isolated guess from extensive disengagement. Future studies should collect item-level guessing or confidence ratings.

Third, the final sample was modest and was not determined by an a priori power calculation. Thirty-three participants completed both sessions, and subdivision into four profiles produced small subgroups. Consequently, the study was not adequately powered for multivariable prediction of profile membership or formal testing of relationships between PSS-4, EI-derived characteristics, baseline capacities, and DTC outcomes.

Fourth, detailed qualitative interpretation focused on two participants showing the largest δ within each profile. Extreme-case sampling facilitates rich description of clearly expressed patterns but does not establish that the observed experiential characteristics are typical of all individuals assigned to that profile.

## 5 Conclusion

This exploratory mixed-method study demonstrates the feasibility of integrating quantitative cognitive-motor performance measures with EI to characterize inter-individual behavior during a cognitive-motor protocol. All major profile patterns were observed, and the qualitative cases illustrated heterogeneous combinations of distress, engagement, strategy, and sensory experience. Although showing the relevance of our methodology, the present data do not permit strong causal or confirmatory conclusions regarding determinants of DT behavior, which are left for future works. The principal contribution of the study is therefore methodological and hypothesis-generating. Larger, prospectively powered studies using counterbalanced designs, equivalent or parallel cognitive-task forms, and trial-level measures of guessing or confidence are needed to determine whether the experiential patterns identified here reliably predict cognitive-motor performance and profile membership.

From an applied and clinical perspective, our dual-tasking protocol thus appears to be a promising tool for probing the interactions between cognition, motor control and psychological factors, and for identifying person-specific profiles that could guide more personalized rehabilitation. Future work should first aim at finding a minimal clinically important difference (MCID) for δ. Obviously indeed, a too small δ should be regarded as an irrelevant DT modification. Having such a MCID would allow to extend our mixed-methods framework to older adults and clinical populations with reduced automaticity or cognitive impairment and to explore how DT interventions might deliberately harness eustress and experiential factors to optimize both gait and cognitive outcomes.

## Data Availability

The datasets presented in this study can be found in online repositories. The names of the repository/repositories and accession number(s) can be found below: OSF repository https://osf.io/jpbdc.
